# Computer-Based Driving in Dementia Decision Tool With Mail Support: Cluster Randomized Controlled Trial

**DOI:** 10.2196/jmir.9126

**Published:** 2018-05-25

**Authors:** Mark J Rapoport, Carla Zucchero Sarracini, Alex Kiss, Linda Lee, Anna Byszewski, Dallas P Seitz, Brenda Vrkljan, Frank Molnar, Nathan Herrmann, David F Tang-Wai, Christopher Frank, Blair Henry, Nicholas Pimlott, Mario Masellis, Gary Naglie

**Affiliations:** ^1^ Department of Psychiatry Sunnybrook Health Sciences Centre Toronto, ON Canada; ^2^ Department of Psychiatry University of Toronto Toronto, ON Canada; ^3^ Department of Research Design and Biostatistics Sunnybrook Research Institute Toronto, ON Canada; ^4^ Institute of Health Policy, Management and Evaluation University of Toronto Toronto, ON Canada; ^5^ Department of Family Medicine McMaster University Hamilton, ON Canada; ^6^ Division of Geriatric Medicine The Ottawa Hospital Ottawa, ON Canada; ^7^ Division of Geriatric Medicine University of Ottawa Ottawa, ON Canada; ^8^ Seniors Mental Health Program Providence Care Kingston, ON Canada; ^9^ Department of Psychiatry Queen's University Kingston, ON Canada; ^10^ School of Rehabilitation Science McMaster University Hamilton, ON Canada; ^11^ Memory Clinic University Health Network Toronto, ON Canada; ^12^ Division of Neurology Division of Geriatric Medicine University of Toronto Toronto, ON Canada; ^13^ Specialized Geriatric Services Providence Care Kingston, ON Canada; ^14^ Division of Geriatric Medicine Queen's University Kingston, ON Canada; ^15^ Clinical Ethics Centre Sunnybrook Health Sciences Centre Toronto, ON Canada; ^16^ Department of Family and Community Medicine University of Toronto Toronto, ON Canada; ^17^ Department of Family and Community Medicine Women's College Hospital Toronto, ON Canada; ^18^ Department of Medicine Sunnybrook Health Sciences Centre Toronto, ON Canada; ^19^ Division of Neurology University of Toronto Toronto, ON Canada; ^20^ Department of Medicine Baycrest Health Sciences Toronto, ON Canada; ^21^ Division of Geriatric Medicine University of Toronto Toronto, ON Canada

**Keywords:** dementia, mild cognitive impairment, automobile driving, decision support systems, clinical

## Abstract

**Background:**

Physicians often find significant challenges in assessing automobile driving in persons with mild cognitive impairment and mild dementia and deciding when to report to transportation administrators. Care must be taken to balance the safety of patients and other road users with potential negative effects of issuing such reports.

**Objective:**

The aim of this study was to assess whether a computer-based Driving in Dementia Decision Tool (DD-DT) increased appropriate reporting of patients with mild dementia or mild cognitive impairment to transportation administrators.

**Methods:**

The study used a parallel-group cluster nonblinded randomized controlled trial design to test a multifaceted knowledge translation intervention. The intervention included a computer-based decision support system activated by the physician-user, which provides a recommendation about whether to report patients with mild dementia or mild cognitive impairment to transportation administrators, based on an algorithm derived from earlier work. The intervention also included a mailed educational package and Web-based specialized reporting forms. Specialists and family physicians with expertise in dementia or care of the elderly were stratified by sex and randomized to either use the DD-DT or a control version of the tool that required identical data input as the intervention group, but instead generated a generic reminder about the reporting legislation in Ontario, Canada. The trial ran from September 9, 2014 to January 29, 2016, and the primary outcome was the number of reports made to the transportation administrators concordant with the algorithm.

**Results:**

A total of 69 participating physicians were randomized, and 36 of these used the DD-DT; 20 of the 35 randomized to the intervention group used DD-DT with 114 patients, and 16 of the 34 randomized to the control group used it with 103 patients. The proportion of all assessed patients reported to the transportation administrators concordant with recommendation did not differ between the intervention and the control groups (50% vs 49%; *Z*=−0.19, *P*=.85). Two variables predicted algorithm-based reporting—caregiver concern (odds ratio [OR]=5.8, 95% CI 2.5-13.6, *P*<.001) and abnormal clock drawing (OR 6.1, 95% CI 3.1-11.8, *P*<.001).

**Conclusions:**

On the basis of this quantitative analysis, in-office abnormal clock drawing and expressions of concern about driving from caregivers substantially influenced physicians to report patients with mild dementia or mild cognitive impairment to transportation administrators, but the DD-DT tool itself did not increase such reports among these expert physicians.

**Trial Registration:**

ClinicalTrials.gov NCT02036099; https://clinicaltrials.gov/ct2/show/NCT02036099 (Archived by WebCite at http://www.webcitation.org/6zGMF1ky8)

## Introduction

### Motor Vehicle Collisions

In 2010, there were 1.24 million fatalities from motor vehicle collisions (MVCs) internationally, representing the eighth leading cause of death, and this is predicted to rise to fifth place by 2030 [1]. It has also been estimated that at least 20 people sustain nonfatal injuries for every MVC fatality [2]. The crash rate per mile driven begins to increase at 65 years [3], and older drivers have the highest fatality rate per mile driven among drivers over the age of 25 years [3]. Although most of the older drivers are safe drivers, various medical conditions may impact their driving ability [3]. The risk of collisions increases with age, and although this increased risk may be largely attributable to those with low mileage, collisions in older adults are more likely to be lethal than in younger adults [4]. In clinical practice, predicting driving safety in this population is very challenging [5].

### Dementia

Dementia refers to a syndrome of chronic and usually progressive cognitive decline caused by changes in the structure and function of the brain. Alzheimer disease (AD) is implicated, either alone or in combination with other causes in more than two-thirds of the cases of dementia in epidemiological and autopsy samples [6,7]. Patients with AD show an inevitable decline in cognition, which ultimately will affect driving abilities over time [8]. Age is the biggest risk factor for AD, with individuals between the ages of 80 to 89 years being 7 times as likely to have AD, and those aged 90 years and older being 38 times as likely to have AD, relative to those between the ages of 70 to 79 years in a community study [9]. It is estimated that there will be 6.7 million older adults with dementia in the United States by 2031 [10] and 1.1 million in Canada by 2038 [11].

### Driving Safety and Dementia

Data from the Canadian province of Ontario also indicate that in 2000, an estimated 34,105 people with AD and related dementias were driving, with the number projected to climb to 98,032 in 2028 [12]. Many patients with mild AD may be safe to drive for some time [8,13,14], and driving cessation in dementia is associated with depression and social isolation as well as mortality [15]. On the other hand, there are significant safety concerns associated with driving in this population. Crash rates may be increased by 2 to 8 times [16,17] in dementia, although several studies have been negative with a failure to control for driving exposure [18], and our most recent systematic review update yielded inconclusive results about this increased crash risk [19]. Patients with dementia have more consistently been demonstrated to have a significantly increased rate of failure when given on-road tests of driving abilities [20-24], with a risk ratio (RR) of 10.77 (95% CI 3.00-38.62) for on-road failure rates among patients with very mild and mild dementia in our recent meta-analysis [19]. Other types of dementia, beyond AD, for example, dementia from cerebrovascular disease, Parkinson disease dementia, Lewy body dementia, and frontotemporal dementia have less predictable impacts on driving ability [8,25-28]. In-office tests have limited ability to predict crashes and on-road test failures in dementia [29-31]. Composite measures of attention, visuospatial skills, global cognition, and especially executive dysfunction are associated with crashes and on-road test failures in part [29,31], but misclassification rates are high and cutoff scores are lacking, limiting their clinical utility [30]. Mild cognitive impairment (MCI) is a condition in which there is concern about a change of cognition, with objective evidence of cognitive impairment, but with preserved independent functioning [32]. MCI is felt to be a risk factor for dementia, but few studies have explored its association with driving safety, and different classification systems for MCI make this a particularly challenging area for physicians [33].

### Medical Reporting on Driving Safety and Dementia

Seven US states and most Canadian provinces have legislation mandating the reporting of medically impaired drivers to transportation administrators [34], and clinical guidelines issued by the Canadian [35] and American Medical Associations [36] emphasize individualized assessments of drivers with dementia. However, these guidelines do not provide concrete suggestions about issues pertaining to reporting in cases of either mild dementia or MCI. Many physicians avoid discussing driving concerns, do not report their patients to transportation administrators, nor advise them on the issue of driving cessation [37-39], at least in part because of concerns over negative impacts on the doctor-patient relationship [37,38].

We conducted an earlier modified Delphi study, Driving and Dementia in Ontario (DADIO) [40,41], in which physician experts in dementia were asked whether or not they would report a patient with mild dementia or MCI to transportation administrators based on 26 hypothetical case scenarios. After 5 iterations, consensus was ultimately obtained for the majority of scenarios, and an algorithm was created to reflect the case scenarios and corresponding expert-derived reporting decisions. In that study, caregiver concern and abnormal performance on the clock drawing test (CDT) [42] accounted for 62% of the variance in reporting such patients. We also found that male physicians were 14% more likely to report than their female counterparts [41].

A multifaceted computer-based knowledge translation intervention was developed using the algorithm developed from the DADIO study as well as qualitative interviews with physicians, caregivers of former drivers with dementia, and transportation administrators. The interviews focused on facilitators and barriers to mandatory reporting and on the algorithm. The intervention, called the Driving in Dementia Decision Tool (DD-DT), also incorporated an updated review of the literature and international guidelines. The DD-DT aims to increase consistency in physician decision-making related to reporting drivers with mild dementia or MCI to transportation administrators [43].

The objective of this study was to evaluate the effects of DD-DT on physicians’ reporting of patients with mild dementia and MCI to transportation administrators, to evaluate its effect on physician recommendations to patients to undergo specialized on-road testing, and to examine its effect on the physicians’ perceptions of the doctor-patient and doctor-caregiver relationship.

## Methods

### Design

We conducted a cluster randomized controlled trial (RCT), in which physicians (the clusters) were randomized to either the DD-DT intervention or a control group. The trial ran from September 9, 2014 to January 29, 2016.

### Intervention

DD-DT and its development are described elsewhere in detail [43]. Briefly, a computer-based clinical decision support system (CCDSS) was created to guide decisions for reporting patients with mild dementia or MCI to transportation administrators (see Multimedia Appendix 1). A training video was embedded in the DD-DT website. When using DD-DT, physicians were asked to input the following variables—patient’s cognitive diagnosis (MCI or mild dementia), history of MVCs in the last 2 years (“driving history”), caregiver or informant concern about the patient’s driving, behavioral or neuropsychiatric disturbances in the patient, level of independence in the performance of activities of daily living and instrumental activities of daily living, and results of in-office cognitive assessment, including the speed of performance on these tests, the patient’s performance on CDT [42], the Mini-Mental State Exam (MMSE) [44] score, or the Montreal Cognitive Assessment (MoCA) [45] score. Input of findings on the Trail Making Test (versions A and B) [46] was considered optional.

Depending on the data input, participants received a recommendation of “Report” to transportation administrators (see Multimedia Appendix 2), “Don’t Report” (see Multimedia Appendix 3), or “No Consensus” (see Multimedia Appendix 4), with the latter recommendation indicating that the data input does not lead to a definitive recommendation, as determined by the DADIO study and algorithm [40]. Additionally, if physicians chose to report the patient, they were directed to prepopulated Ministry of Transportation of Ontario reporting forms within the computer-based DD-DT intervention (see Multimedia Appendix 5) to facilitate the reporting process. If they chose not to issue a report despite an algorithmic recommendation to do so, physicians were asked to document their rationale. Information packages for patients and caregivers were also mailed to participants, so physicians had them available to give to the patients regardless of the decision to report to transportation administrators. The package included a copy of the driving and dementia toolkit for patients and caregivers [47], which includes information on coping with grief, how to contact the local branch of the Alzheimer society, suggestions about how to initiate conversations regarding driving safety with persons with dementia, a list of local Ministry-approved specialized driving assessment centers, and a list of alternative sources of transportation in the participant’s region. Thus, the DD-DT intervention encompassed the computer-based decision support system, a specialized reporting form, and a mailed information package.

### Control

Physicians in the control group were instructed to input the same data onto the website as the DD-DT intervention group. However, the control group version of the DD-DT did not generate an algorithm-based reporting decision. Neither a patient/caregiver information package nor a Ministry of Transportation reporting form was provided for the control group. Instead, the physician received a generic prompt reminding them of the mandatory reporting legislation for patients in Ontario with conditions that may affect driving.

### Participants (Physicians)

Information about the study was distributed by email using national physician organizations that represent Geriatric Medicine and Geriatric Psychiatry, as well as groups of family physicians specializing in Care of the Elderly or dementia care. The investigators also contacted members of their respective disciplines to further facilitate recruitment. We also advertised in 3 continuing medical education journals and at a primary care conference on May 7 to May 9, 2015. To be included, physicians had to have access to a computer and printer in the clinical area where they saw patients. They also needed to confirm they saw at least 12 new patients per year with mild dementia or MCI. Participants in the DADIO project were eligible to participate, provided that they did not attend a June 2012 study meeting with investigators, where they would have been exposed to the algorithm, which informed the DD-DT tool. All participants were in Ontario, Canada, a jurisdiction with mandatory requirements on medical practitioners to report individuals suffering from any conditions that may make it dangerous to operate a motor vehicle [48].

### Assessments (Patients)

Physicians were instructed to use the tool only with patients aged 60 years and over, who had mild dementia or MCI, and who continued to drive. Participating physicians were instructed not to use the tool for patients with moderate or severe dementia or those for whom the most responsible cause of the cognitive impairment was a primary psychiatric disorder, delirium, or alcohol or substance use.

### Outcomes

After using the tool, physicians in both the intervention and control groups indicated whether or not they decided to report the patient to the Registrar of the Ministry of Transportation of Ontario (referred to as transportation administrators in this manuscript). They also indicated if a specialized road test was recommended for the patient in question. As the main goal of the tool was to enhance the physicians’ reporting of patients deemed to be at significant risk of unsafe driving, the primary outcome of this study was the number of reports made to the local transportation administrators concordant with recommendations of the DD-DT algorithm (true positives). The primary outcome was the proportion of all assessments in which participants made an algorithm-concordant report (true positives/all assessments). Secondary outcomes included “any reports” filed to the transportation administrators, whether or not concordant with the DD-DT algorithm (true positives+false positives/all assessments), and recommendations for a specialized on-road test. Finally, we explored the participants’ perception of the doctor-patient relationship and the doctor-caregiver relationship after each assessment. A 5-point Likert scale was used with scores ranging from −2 (“Extremely negative”) to +2 (“Extremely positive”), with 0 representing “Neither negative nor positive.” We also asked about their perception of how any pressure they felt from family members played into their decision, with scores ranging from 0 to +4 and 0 representing “Not at all” and 4 representing “A great deal.”

### Mediators

We also measured physician-related factors that may have predicted reporting, including physician years in practice, the sex of the physician, the type of practice (family physician or specialist), whether the physician practiced in an urban community (based on their postal code), and whether their practice was hospital-based or community-based. Participants also completed the Risk-Taking Scale (RTS) that assessed their level of comfort with risk-taking versus risk-aversion, along with the Stress from Medical Uncertainty Scale (SUS), both of which are described by Pines [49] as potential physician-related mediating factors.

Patient-related mediating factors that were analyzed included the patient’s age, whether the caregiver was concerned about the patient’s driving (coded as yes, no, or uncertain), and whether the CDT was abnormal, based on the Mini-Cog scoring system [50]. Other potential mediators, including patient diagnosis and scores on other cognitive tests, were measured but not included in the multivariable analysis because of the limitations posed by the sample size.

### Randomization

We used a cluster RCT design, in which each physician participant was considered as a “cluster.” A statistician-generated randomization sequence was used to ensure equal probability of each physician participant being assigned to the intervention or control group, in a 1:1 ratio. Randomization was stratified based on sex in permuted blocks of 4 and 8 to ensure equal numbers of males and females in the intervention and control groups. Physicians who agreed to participate and provided informed consent were provided with a link to access the Web-based decision tool. Given the nature of the intervention, participants were aware of group assignment and, as such, blinding was impossible.

### Analysis

Univariate analysis of variance, chi-square, and Fisher exact tests were used to compare physician and patient demographic and clinical variables between the intervention and control groups, and between those randomized who used the tool versus those who did not use the tool. We also used these univariate tests to compare the same variables between patients reported in concordance with the DD-DT algorithm (true positives) and those for whom a per-algorithm report was not issued (true negatives and false negatives). For these analyses, a tool recommendation of “No Consensus” led to a classification of concordance with the algorithm, regardless of the physician’s subsequent decision and action. Then, a 2-sample, 2-sided test of proportions compared the primary outcome, reporting percentage of those deemed to be of concern (ie, “reporting concordant with algorithm”), between groups. A generalized estimating equation model with a logit link function was run to compare reporting between groups as well as physician and patient mediating factors, which were adjusted for the clustered nature of the data, assuming an exchangeable correlation structure. Before analysis, multicollinearity among the predictor variables was assessed using tolerance statistics, with a value of less than 0.4 as the cut point. If multicollinearity was found, then only 1 member of a correlated set of variables was retained for the final model. Bivariate analyses were carried out on the remaining set of mediators in relation to the outcome. The final model included those with *P* values <.20 on these analyses. A similar analysis was conducted for the secondary outcomes of “any reports to transportation administrators” and “any road-test recommendations” (ie, regardless of whether these were concordant with the DD-DT algorithm). Ordinal regression models were run to examine doctor-patient relationship and doctor-caregiver relationship in relation to the predictors of interest.

### Sample Size Calculation

We anticipated a base rate of reporting to transportation administrators of 13.0% (13/100) in the control group based on data from academic family practice [39]. We conducted an informal poll with 6 of the knowledge-users involved in the design of this study, enquiring about what they would view as the “minimal clinically important difference” as a trade-off for the main cost of time spent on assessment. This yielded a mean absolute difference of 12.2% (SD 3). We based the sample size on a more conservative absolute difference of 10% (ie, 13% vs 23%), in line with a recent comprehensive review of multifaceted knowledge translation interventions [51]. We planned for a sample of 36 clusters (ie, physicians) in both the intervention and control groups, and assumed an average of 7 patient assessments per physician. Using an unpooled 2-sided Z test, an alpha of .05, and an intracluster correlation coefficient of .02, a sample size of 252 assessments in each group would yield 80% power to detect an absolute difference of 10% between groups. We anticipated a 33.0% (33/100) attrition rate and, as such, planned to recruit 54 participants per arm. A maximum of 12 assessments were allowed per participant, and data were censored after that number had been reached.

### Changes to the Study Protocol

We initially included physicians who indicated they typically saw at least 12 new patients per year with mild dementia or MCI; however, we quickly realized that many potential participants did not have this volume of patients with MCI or mild dementia who were still driving. We, therefore, subsequently removed this minimum volume requirement.

Several steps were taken to keep study participants engaged and to encourage them to remember to use the online tool when seeing patients with mild dementia or MCI. We sent participants in both groups up to 4 newsletters via email over the course of the study (with later recruited participants receiving fewer than 4 letters), which provided updates on research about driving and dementia, but deemed to be unlikely to influence the participants’ decisions regarding whether or not to report their patients to driving administrators, or to recommend a road test (see Multimedia Appendix 6). We also invited participants to implement a chart flag system, which provided a visual reminder to use the online tool when seeing patients with mild dementia or MCI. In addition, we increased the honorarium to participants from Can $20 to $40 per use midway through the course of the study in an effort to encourage use of the tool. Finally, we gave control group participants an opportunity to use the intervention version of the tool following the conclusion of the study.

### Ethics

The study was approved by the Research Ethics Office of Sunnybrook Health Sciences Centre, #269-2013, and written informed consent was obtained for all participants.

## Results

### Participants and Evaluations

**Figure 1:**
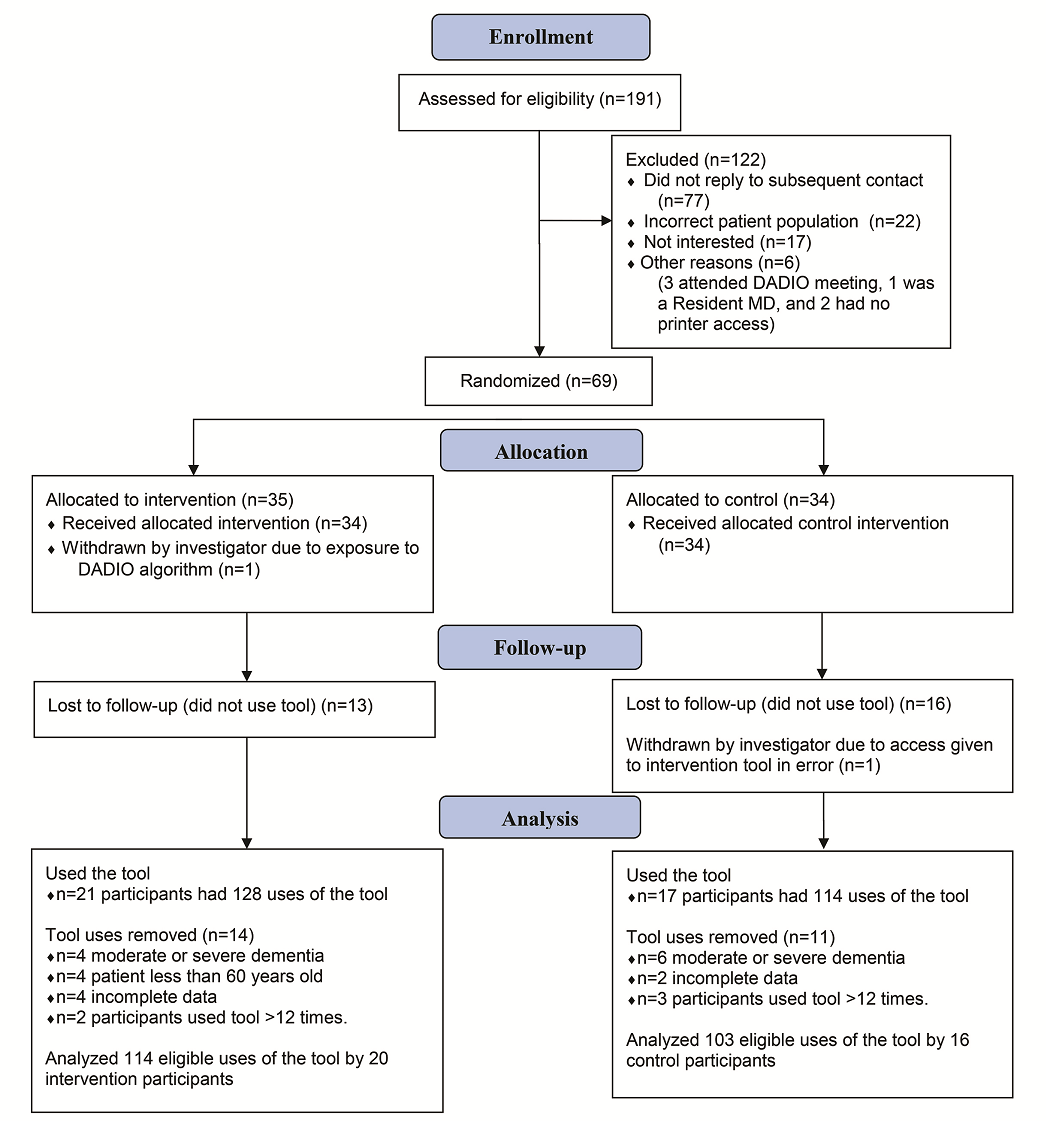
Consolidated Standards of Reporting Trials (CONSORT) diagram of recruitment, randomization, allocation, follow-up, and analysis.

A total of 191 physicians expressed an interest in participating in this study. Of these, 77 did not reply to follow-up emails after initial contact, 22 were ineligible because they did not meet patient population eligibility criteria, 17 were not interested, and 6 were ineligible for other reasons (3 had been exposed to the algorithm that formed the basis of the decision tool at the DADIO study meeting, 2 did not have a printer in the area where they saw patients, and 1 was a resident physician still in training). A total of 69 physicians were enrolled in the study and 35 were randomized to the intervention group and 34 to the control group. Of the 35 physicians in the intervention group, only 20 used DD-DT at least once, with a total of 114 eligible individual patient assessments in this group. Of the 34 physicians in the control group, only 16 used the control tool at least once, with a total of 103 eligible patient assessments in that group (see Figure 1).

Participants who were randomized but did not use the tool were no different from those who did use the tool in terms of group assignment, gender, type or location of practice, or years in practice. However, those who were randomized but did not use the tool scored higher on RTS than those who did use the tool, indicating a higher tolerance of risk (*F*_1,60_=4.702, *P*=.03, and a nonsignificant tendency to score lower on SUS (*F*_1,60_=3.765, *P*=.057), indicating less stress from uncertainty (see Multimedia Appendix 7).

There were no significant differences in the intervention and control groups in the physician sex, years in practice, type of practice or location of practice, or on the RTS or SUS scales (Table 1). The patients assessed by the physicians in each group were similar in age, sex, history of collisions, and other measured clinical features (Table 2). Overall, 117 out of 217 patients assessed had MCI and 100 had dementia; in 16 of the latter cases, the physician was uncertain if the patient had moderate dementia. Table 3 shows etiology of MCI or dementia for patients assessed.

### Reporting to Transportation Administrators Concordant With the Tool Recommendation

In the univariate analysis, the proportion of patients reported to transportation administrators concordant with the tool recommendation did not differ statistically between the intervention and the control groups, with a report issued in 50.0% (57/114) of the assessments in the intervention group and 48.5% (50/103) of the assessments in the control group. Raw data on the concordance between tool recommendation and participant action are provided in Multimedia Appendix 8. There were no differences in the measured physician-related variables between patients reported concordant with the recommendation versus other assessments in which a recommendation-concordant report was not issued (Tables 4 and 5); however, all of the measured patient clinical variables, except patient age and rural or urban status, were associated with reports issued concordant with the recommendation (Tables 6 and 7). Overall, there was concordance between the tool recommendation and subsequent physician decision in 199 out of the 217 cases, including 59 intervention group cases and 37 control group cases in which the concordance was due to a tool recommendation of “No Consensus.”

**Table 1:** Between-group comparisons for the participants.

Variable	Control group (n=16)	Intervention group (n=20)	Statistic		
			Chi-square (degrees of freedom)	F test (degrees of freedom)	*P* value
Physician sex (male), n (%)	8 (50)	5 (25)	2.4 (1)	—	.12
Physician years in practice, mean (SD)	13.44 (10.33)	14.58 (9.51)	—	0.118 (1,35)	.73
Type of practice (family medicine rather than specialty), n (%)	6 (38)	10 (50)	0.6 (1)	—	.45
Geographic location of practice (rural rather than urban), n (%)	0 (0)	2 (10)	—	—	.49^a^
Hospital-based practice (hospital-based rather than community-based practice), n (%)	11 (69)	12 (60)	0.3 (1)	—	.59
Risk taking scale, mean (SD)	17.56 (4.15)	16.50 (5.17)	—	0.446 (1,35)	.51
Stress from medical uncertainty scale, mean (SD)	42.13 (9.86)	42.15 (11.37)	—	0.0001 (1,35)	.99

^a^Fisher exact test.

**Table 2:** Between-group comparisons for the patients assessed by the participants.

Variable	Control group	Intervention group	Statistic		
			*t* test (degrees of freedom)	*Z* value	*P* value
Patient age in years^a^, mean (SD)	78.12 (7.62)	77.73 (7.16)	0.24 (34)	—	.81
Patient sex^a^ (male), n (%)	62 (60.2)	62 (54.4)	—	0.74	.46
Patient location^a^ (rural), n (%)	15 (14.6)	30 (26.3)	—	0.96	.34
Mild cognitive impairment^a^ (ie, not mild dementia), n (%)	54 (52.4)	63 (55.3)	—	−0.28	.78
Caregiver concern^a,b^, n (%)	37 (35.9)	36 (31.6)	—	−0.66	.51
Motor vehicle collisions^a,b^, n (%)	11 (10.7)	14 (12.3)	—	0.41	.68
Abnormal clock drawing test^a,c^, n (%)	62 (60.2)	61 (53.5)	—	−0.65	.51
MMSE^d^, mean (SD)	24.07 (3.70)	25.74 (3.56)	−1.67 (18)	—	.11
MoCA^e^, mean (SD)	20.77 (3.84)	20.42 (3.87)	0.44 (32)	—	.66
Behavioral or neuropsychiatric disturbances^a,b^, n (%)	25 (24.3)	21 (18.4)	—	−0.85	.39
Cognitive slowing^a^, n (%)	32 (31.1)	33 (29.0)	—	−0.08	.94
Abnormal Trails B result^f,g^, n (%)	33 (62.3)	51 (76.1)	—	1.60	.11

^a^Based on 217 observations.

^b^For caregiver concern, motor vehicle collisions, and behavioral or neuropsychiatric disturbances, a response of “unknown” was treated as “no” and combined with “no” for analyses.

^c^We used the Mini-Cog algorithm for scoring abnormal performance on the CDT. Specifically, “The CDT is considered normal if all the numbers are present in the correct sequence and position, and the hands readably display the requested time.” [52].

^d^MMSE: Mini-Mental State Exam. Based on 75 observations.

^e^MoCA: Montreal Cognitive Assessment. Based on 182 observations.

^f^Abnormal Trails B result defined as completion time of >3 min or ≥3 errors [53].

^g^Based on 120 observations.

**Table 3:** Etiology of mild cognitive impairment (MCI) or dementia for patients assessed.

Type of disorder	Patients^a^, n (%)
MCI^a^	86 (39.6)
Vascular cognitive impairment	22 (10.1)
Other MCI	9 (4.1)
Alzheimer disease	47 (21.7)
Vascular dementia	5 (2.3)
Mixed dementia	39 (18.0)
Lewy body dementia	1 (0.5)
Frontotemporal dementia	1 (0.5)
Dementia due to brain injury	1 (0.5)
Dementia type not yet diagnosed	5 (2.3)
Other dementia	1 (0.5)

^a^Based on 217 observations.

^b^MCI: mild cognitive impairment.

**Table 4:** Physician predictors of reports concordant with the tool recommendation (dichotomous variables).

Variable	Number of reports concordant with the tool recommendation, n (%)	Statistic	
		Chi-square (degrees of freedom)	*P* value
Intervention group	57 (50.0)	—	—
Control group	50 (48.5)	0.05 (1)	.83
Male physicians	37 (52)	—	—
Female physicians	70 (47.9)	0.35 (1)	.57
Rural physicians	5 (38)	—	—
Urban physicians	102 (50.0)	0.7 (1)	.42
Hospital-based practice	83 (49.7)	—	—
Community-based practice	24 (48)	0.05 (1)	.83
Family physicians	35 (45)	—	—
Specialists	72/140 (51.4)	0.7 (1)	.40

**Table 5:** Physician predictors of reports concordant with the tool recommendation (continuous variables).

Variable	Reported concordant with algorithm (n=107), mean (SD)	Other assessments^a^ (n=110), mean (SD)	Statistic	
			*F* test (degrees of freedom)	*P* value
Physician years in practice	13.813 (9.31)	13.368 (8.49)	0.135 (1,216)	.71
Risk taking scale	16.75 (6.06)	16.77 (4.57)	0.001 (1,216)	.97
Stress from medical uncertainty scale	41.78 (11.04)	41.06 (11.16)	0.223 (1,216)	.64

^a^For this category, in 110 assessments, there were 92 cases in which physicians followed the algorithm recommendation not to report to transportation administrators, 2 cases in which physicians reported when the tool recommended not to do so, and 16 cases in which physicians did not report when the tool recommended a report.

**Table 6:** Patient predictors of reports concordant with the tool recommendation (dichotomous variables).

Variable	Number of reports concordant with the tool recommendation, n (%)	Statistic^a^	
		Chi-square (degrees of freedom)	*P* value
Male patients	54 (43.5)	—	—
Female patients	53 (57)	3.8 (1)	.05
Rural patients	20 (44)	—	—
Urban patients	87 (50.6)	0.6 (1)	.46
Mild dementia	74 (74.0)	—	—
Mild cognitive impairment	33 (28.2)	45.2 (1)	<.001
Caregiver concern	56 (77)	—	—
No caregiver concern	51 (35.4)	33.1 (1)	<.001
History of collisions	21 (84)	—	—
No history of collisions	86 (44.79)	13.6 (1)	<.001
Abnormal clock drawing test^a^	82 (66.7)	—	—
Normal clock drawing test	25 (27)	34.2 (1)	<.001
Behavioral or neuropsychiatric disturbances	37 (80)	—	—
No behavioral or neuropsychiatric disturbances	70 (40.9)	22.6 (1)	<.001
Cognitive slowing	49 (75)	—	—
No cognitive slowing	58 (38.1)	25.2 (1)	<.001
Abnormal Trails B result^b^	58 (69)	—	—
Normal Trails B result	2 (6)	40.6 (1)	<.001

^a^We used the Mini-Cog algorithm for scoring abnormal performance on clock drawing test (CDT). Specifically, “The CDT is considered normal if all the numbers are present in the correct sequence and position, and the hands readably display the requested time.” [52].

^b^Abnormal Trails B result defined as completion time of >3 min or ≥3 errors [53]. n=120 cases included the Trails B data.

**Table 7:** Patient predictors of reports concordant with the tool recommendation (continuous variables). MMSE: Mini-Mental State Exam; MoCA: Montreal Cognitive Assessment.

Variable	Reported per algorithm (n=107), mean (SD)	Other assessments^a^ (n=110), mean (SD)	Statistic	
			*F* test (degrees of freedom)	*P* value
Patient age in years	78.60 (6.98)^b^	77.25 (7.70)^c^	1.835 (1,216)	.18
MMSE^d^	24.14 (4.05)^e^	25.97 (3.10)^f^	4.901 (1,74)	.03
MoCA^g^	19.03 (3.67)^h^	22.17 (3.36)^i^	36.015 (1,181)	<.001

^a^For other assessments, there were 92 cases in which physicians followed the algorithm recommendation not to report to transportation administrators, 2 cases in which physicians reported when the tool recommended not to do so, and 16 cases in which physicians did not report when the tool recommended a report.

^b^n=107 observations.

^c^n=110 observations.

^d^MMSE: Mini-Mental State Exam.

^e^n=36 observations.

^f^n=39 observations.

^g^MoCA: Montreal Cognitive Assessment.

^h^n=92 observations.

^i^n=90 observations.

There were 8 intervention group cases in which the tool recommended reporting to transportation administrators, but the physician did not issue a report. The rationales for disregarding the tool recommendation included a perceived need for further assessment before reporting educational or medical factors playing a role in low cognitive scores, among others (see Multimedia Appendix 9).

As all tolerance values for the multicollinearity assessment were greater than 0.4, we did not exclude any variables. In the multivariable analysis, the effect of group was not significant (odds ratio [OR]=1.1, 95% CI 0.4-3.0, *P*=.85). Controlling for the effects of group, patient age, participant sex, participant rural or urban status, participant type of practice, participant years in practice, participant RTS scores, participant SUS scores, and clock-drawing abnormalities, the presence of caregiver concern about driving safety was associated with an increase of physician reporting in accordance with the DD-DT algorithm (OR 5.8, 95% CI 2.5-13.6, *P*<.001). Similarly, the presence of clock-drawing abnormalities, controlling for caregiver concern and the variables mentioned in the immediately preceding analysis, was associated with an increased likelihood of such reports (OR 6.1, 95% CI 3.1-11.8, *P*<.001). In the same model, SUS fell just short of statistical significance such that for each 1 unit increase, the odds of issuing such reports were increased (OR 1.04, 95% CI 1.00-1.09, *P*=.06).

### Any Reports to the Transportation Administrators

Similar results were found for the analysis of “any reports” issued to transportation administrators, regardless of whether or not these were concordant with the tool recommendation. Physicians in the intervention group reported 50.9% (58/114) patients assessed, compared with those in the control group who reported 49.5% (51/103) patients. Although group was not significant (OR 1.1, 95% CI 0.4-2.8, *P*=.90), a multivariable analysis controlling for the same variables described for the primary analysis, filing “any report” was significantly associated with the presence of caregiver concern about driving (OR 5.2, 95% CI 2.3-12.0, *P*<.001) and clock-drawing abnormalities (OR 5.4, 95% CI 3.0-9.9, *P*<.001).

### Recommendations for Specialized On-Road Testing

Recommendations for specialized on-road testing were issued by physicians in the intervention group for 32.4% (37/114) patients assessed compared with 33.0% (34/103) patients assessed by physicians in the control group (Z=0.70, *P*=.48). In this case, recommendations for testing were associated with the presence of caregiver concern about driving (OR 2.24, 95% CI 1.17-4.28, *P*=.01) and clock-drawing abnormalities (OR 2.26, 95% CI 1.12-4.53, *P*=.02) using the same model as the prior analyses.

### Impact on the Physician’s Relationship With the Patient and Caregiver

After using the tool, the physicians indicated negative relationships (ie, scores of −2 or −1) with patients in 21.0% (45/215) of assessments, and with caregivers in only 7.0% (15/216) of assessments. There was no significant difference between the intervention or control groups in the physicians’ perceptions of their relationship with the patient or caregiver, controlling for the decision to report, the presence of caregiver concern about driving, or the presence of clock-drawing abnormalities. However, the filing of a report (controlling for group, caregiver concern, and clock-drawing abnormalities) was significantly associated with a lower likelihood of a perceived good relationship with the caregiver (OR 0.34, 95% CI 0.19-0.62, *P*=.01) and with the patient (OR 0.23, 95% CI 0.12-0.43, *P*<.001).

## Discussion

### Principal Findings

We found that use of a multifaceted DD-DT in a Canadian province with mandatory reporting legislation did not increase the likelihood of physician reporting of patients with MCI or mild dementia to transportation administrators, as compared with a legislation reminder of the legislation. Other researchers have assembled algorithms, pathways, or educational approaches to guide clinicians in assessing and reporting to authority drivers with dementia [54-56]. Such approaches have been found to improve physician knowledge and confidence [54,56], and a decision aid geared at patients with dementia, rather than their physicians, reduced decisional conflict in an uncontrolled pilot study [57]. However, this is the first study to assess the impact of these interventions on actual reporting of patients to transportation administrators. Our between-group differences were not clinically meaningful, and a post-hoc sample size calculation indicates that 39,240 assessments in each group would be required to find the difference that we observed with 80% power and a 2-sided alpha of .05.

It is unclear why DD-DT did not increase reporting rates to transportation administrators, but there are several possibilities. We found a much higher reporting rate in the control group than we had anticipated based on earlier work in academic family practice [39] or in our prior study with hypothetical cases in which there was consensus to report just over one-quarter of patients with MCI or mild dementia [40]. It is likely that the effect of being observed in a research study (ie, the “Hawthorne” effect) increased the reporting rate substantially beyond what we had anticipated.

We initially planned to recruit more family physicians than specialists. Specialists, and indeed highly specialized family physicians such as those who participated in this study, may intuitively and implicitly use reporting-related heuristics by virtue of their training and extensive experience with such patients [58], and thereby be less likely to incrementally benefit from DD-DT. Indeed, there is some RCT evidence that more robust clinical outcomes are seen when CCDSSs are used by generalists [59-61], and observational evidence that generalists may be more likely to use best-practice algorithms than specialists [62]. Physician behavior is difficult to change, and to some extent, fears of malpractice suits may drive behavior [63]. Malpractice-related concerns may be particularly salient with regard to not reporting potentially medically-impaired drivers to transportation administrators, and specialists may feel that it is important for them to make such reports to preserve patients’ relationship with their primary care providers [34,37,64]. Previously documented physician concerns about the impact of reporting on the doctor-patient relationship [34,37,64] were confirmed empirically in this study. We did not, however, confirm earlier findings pertaining to physician-related predictors of reporting, such as years in practice [65], physician sex, or self-rated perceived stress from medical uncertainty [41].

Clinical predictors were robustly associated with reporting to transportation administrators. Specifically, concern by caregivers and abnormality on a CDT were found to sway physician behavior, above and beyond the effect of randomized group assignment in our multivariable analysis. A lower MMSE was predictive of reporting to the licensing authority in a large-scale Swedish registry study in which only 9% of 5113 patients with dementia were reported [66]. In our earlier Delphi study [40] in which we presented hypothetical cases, and in this study with real patients, physician reporting was highly tied to cognitive findings and caregiver concerns. Although studies have documented low agreement between physicians’ predictions and on-road results [67,68], when faced with uncertainty about patients with mild dementia in jurisdictions with mandatory reporting, physicians appear to use their judgment about the clinical picture in deciding whether to file these reports. Caregiver concern about driving ability, in particular, is a challenging area for clinicians. When present, it has been recommended as an important consideration and marker for the need for driving assessment, but the absence of caregiver concern is considered less predictive or helpful [69]. Caregiver concerns were associated with road-test outcomes in 2 recent studies of cognitively impaired drivers [70,71]. However, in one of those studies, the caregiver concerns were only correlated with on-road and naturalistic driving outcomes when the caregiver was an adult child (mostly female in that study), but when the caregiver was a spouse (mostly male), better ratings of driving ability were counter-intuitively associated with worse driving performance [70]. Although our univariate analysis showed that abnormal performance on the CDT led physicians to issue a report to transportation administrators, a prior study showed that various scoring systems of the CDT had limited predictive ability of impairment of on-road test performance among those with mild AD and healthy controls [72]. Similarly, Trails B, which was associated with reporting in the univariate analysis, has been shown in a number of studies to be associated with on-road driving performance in dementia, but with limited predictive ability [73], and limitations in the evidence base.

### Strengths of This Study

A review of RCTs of CCDSSs and Knowledge Management systems in 2012 found that less than 20% of 148 studies assessed clinical outcomes [74]. Our study had some important strengths when considering the evaluation of CCDSSs, building in features that have been found to be associated with more successful outcomes in RCTs of CCDSSs [75]. We used a cluster randomization design of physicians to the intervention and control conditions. Physicians in both of these groups were required to enter clinical data about the patients assessed, equalizing the Hawthorne effect by adding a control. We also circumvented the known effects of using checklists [76,77] on physician behavior by requiring clinical data entry in both groups. We randomized the physician rather than the patient to circumvent contamination bias, in which the physician learns the tool with an intervention patient and applies it via “contamination” to a control patient [76]. We stratified our randomization by physician sex, which was found to be an important predictor in our earlier study. We required physicians to provide a reason for their decision if it was discordant with the recommendation of the DD-DT.

### Limitations of This Study

There are some limitations to our work that should be considered. First, there were high levels of nonuse or low use of the tool by the participants enrolled in our study, similar to a naturalistic observation study of CCDSS in primary care for heart failure [78]. We may have found more use of the tool and more between-group differences had we embedded the DD-DT into the physicians’ existing electronic medical records and work-flow procedures of each clinic [74,79], and explicitly provided the justification of the decision support with research evidence. Although there were few differences between those physicians who did and did not use the tool, those who did not use the tool tended to have a higher tolerance of risk and lower stress from medical uncertainty. Second, because we had a large number of cases in which the DD-DT produced a No Consensus recommendation, the number of cases counted as concordant with the algorithm was higher than expected. Third, our results may not be representative of doctors and patients in rural settings, as we included very few of these. Fourth, the patients assessed were heterogeneous with respect to etiology of MCI or dementia, and in a minority of cases, physicians were not completely sure that the dementia was of mild severity. Fifth, we did not incorporate the Trail Making B test into the multivariable analysis, even though it was predictive in the univariate analysis. The rationale was that the Trail Making B test was optional for participants, and only 56% of participants used this as part of their assessment. Furthermore, the sample size precluded adding more than our a priori variables to the multivariable model. Sixth, the results may not generalize to jurisdictions in which there is discretionary rather than mandatory reporting legislation for medically impaired drivers, or to jurisdictions with mandatory reporting legislation that specifically requires reporting of individuals with dementia. Finally, the study results may not apply to physicians who see small numbers of patients with MCI and mild dementia, and have limited expertise in assessing driving risk in patients with cognitive impairment. The tool may be more useful to such physicians than it was in our sample with more expert physicians.

We confirmed that in a jurisdiction with mandatory reporting of medically impaired drivers, physicians base their decision to report concerns about the driving of patients with mild dementia or mild cognitive impairment on caregiver concerns and abnormal clock drawing. However, we did not find that DD-DT increased these reports beyond a simple reminder about the legislation. We also confirmed a negative impact of reporting on the doctor-patient relationship, as perceived by the physician. A preliminary analysis of the qualitative data shows that in general, family physicians had more positive views of the tool than specialist physicians, and some highlighted barriers were identified, including the lack of integration with electronic medical records and the fact that the DD-DT could not incorporate certain contextual nuances. It will be important to understand further the barriers and facilitators of using DD-DT, and a more extensive qualitative analysis of interviews conducted with those physicians who participated in the intervention group, as well as a group of other health care professionals, is under way.

## Appendix

Multimedia Appendix 1 Screenshot of the Driving in Dementia Decision Tool study website home page.

Multimedia Appendix 2 Screenshot of recommendation to report patient to transportation administrators.

Multimedia Appendix 3 Screenshot of recommendation not to report patient to transportation administrators.

Multimedia Appendix 4 Screenshot of no consensus recommendation.

Multimedia Appendix 5 Sample Ministry of Transportation of Ontario reporting form.

Multimedia Appendix 6 References contained in newsletters to DD-DT study participants.

Multimedia Appendix 7 Comparisons between enrolled participants who used the tool and those who did not.

Multimedia Appendix 8 Tool recommendations compared with participant action.

Multimedia Appendix 9 Rationale for "Do not Report" by 6 intervention group participants when tool recommended that the patient be reported.

Multimedia Appendix 10 CONSORT-EHEALTH checklist (V 1.6.1).

## Abbreviations

AD: Alzheimer disease
CCDSS: computer-based clinical decision support system
CDT: Clock Drawing Test
DADIO: Driving and Dementia in Ontario study
DD-DT: Driving in Dementia Decision Tool
MCI: mild cognitive impairment
MMSE: Mini-Mental State Exam
MoCA: Montreal Cognitive Assessment
MVC: motor vehicle collisions
OR: odds ratio
RCT: randomized controlled trial
RTS: Risk-Taking Scale
SUS: Stress from Medical Uncertainty Scale
