# Supplementary figures and images for: Computer-Based Driving in Dementia Decision Tool With Mail Support: Cluster Randomized Controlled Trial
Source: J Med Internet Res. 2018 May 25;20(5):e194. doi: 10.2196/jmir.9126 (PMC5993977; doi:10.2196/jmir.9126)

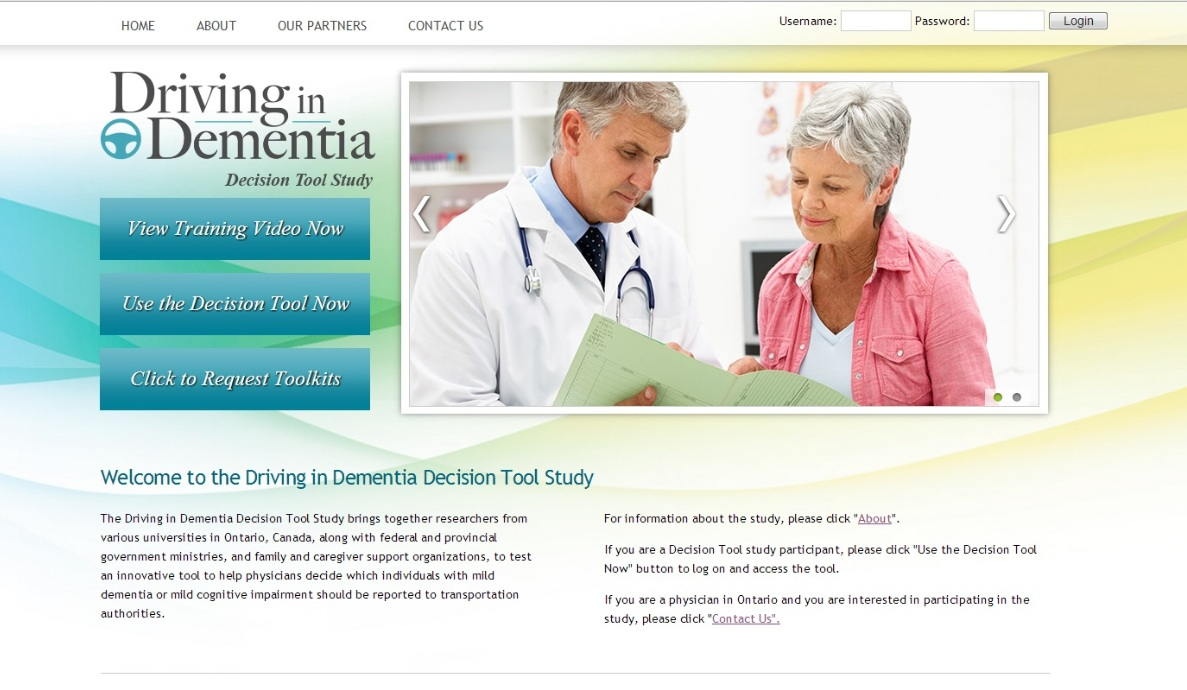

Supplement: Multimedia Appendix 1 [file jmir_v20i5e194_app1.png]

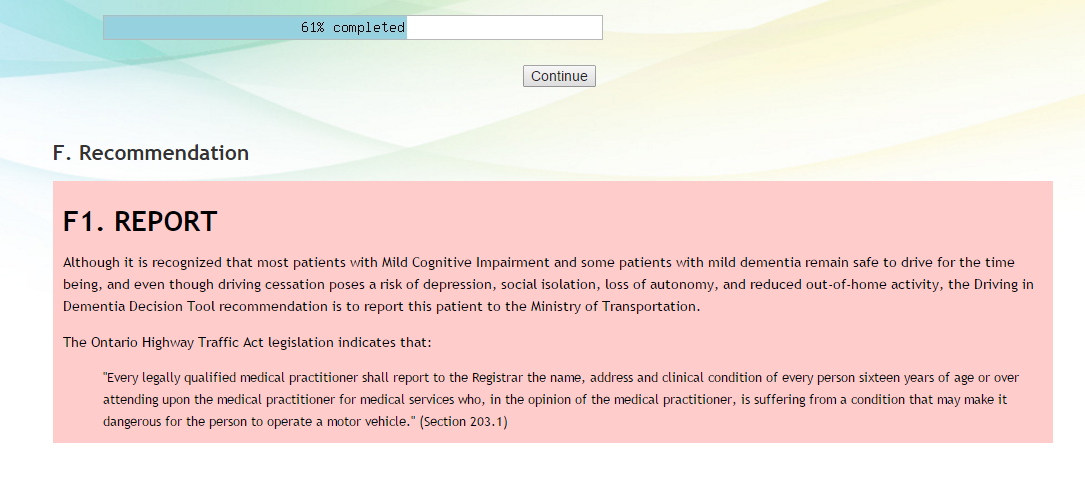

Supplement: Multimedia Appendix 2 [file jmir_v20i5e194_app2.png]

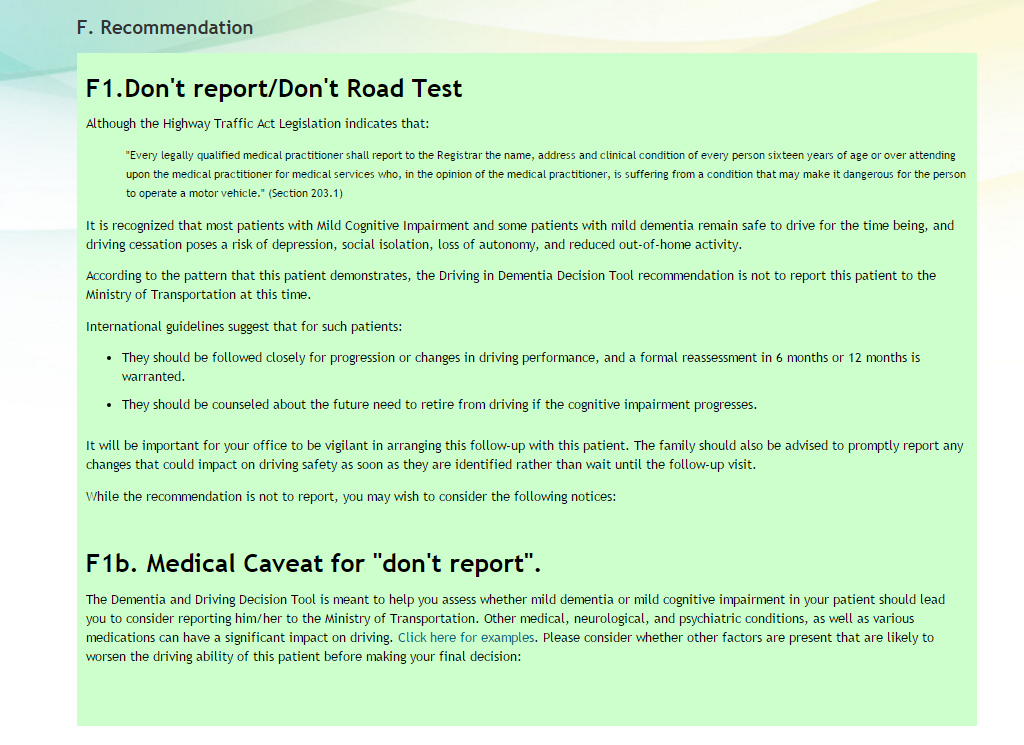

Supplement: Multimedia Appendix 3 [file jmir_v20i5e194_app3.png]

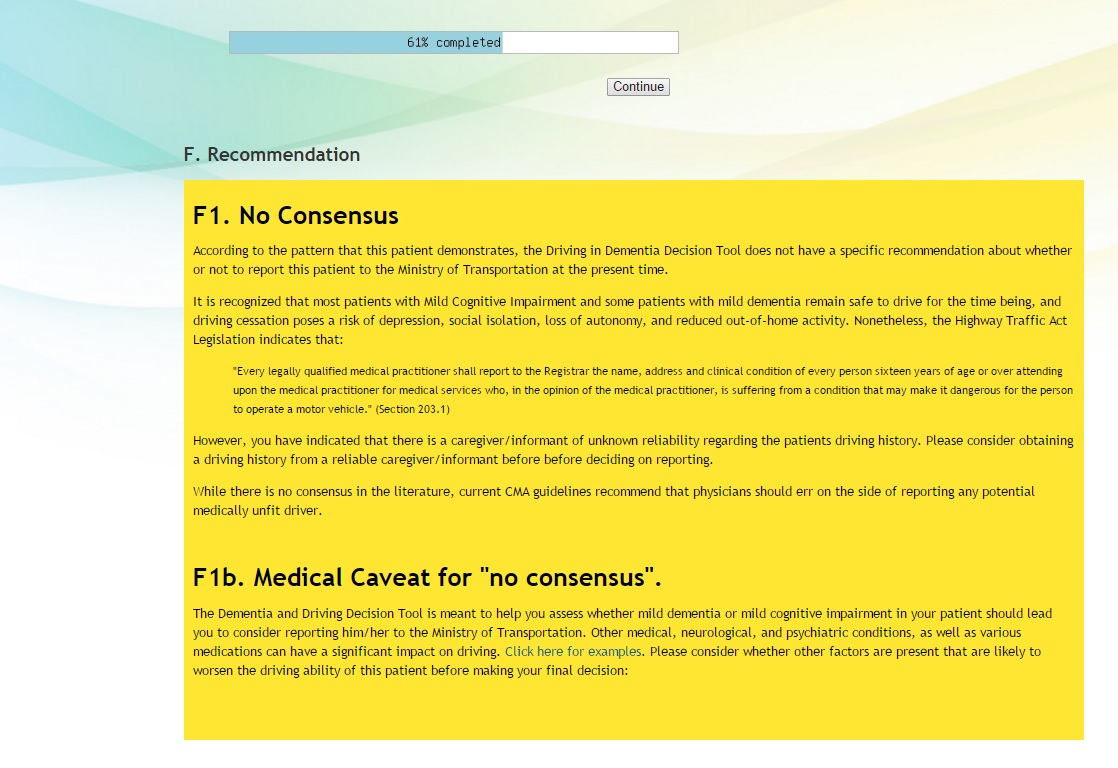

Supplement: Multimedia Appendix 4 [file jmir_v20i5e194_app4.png]

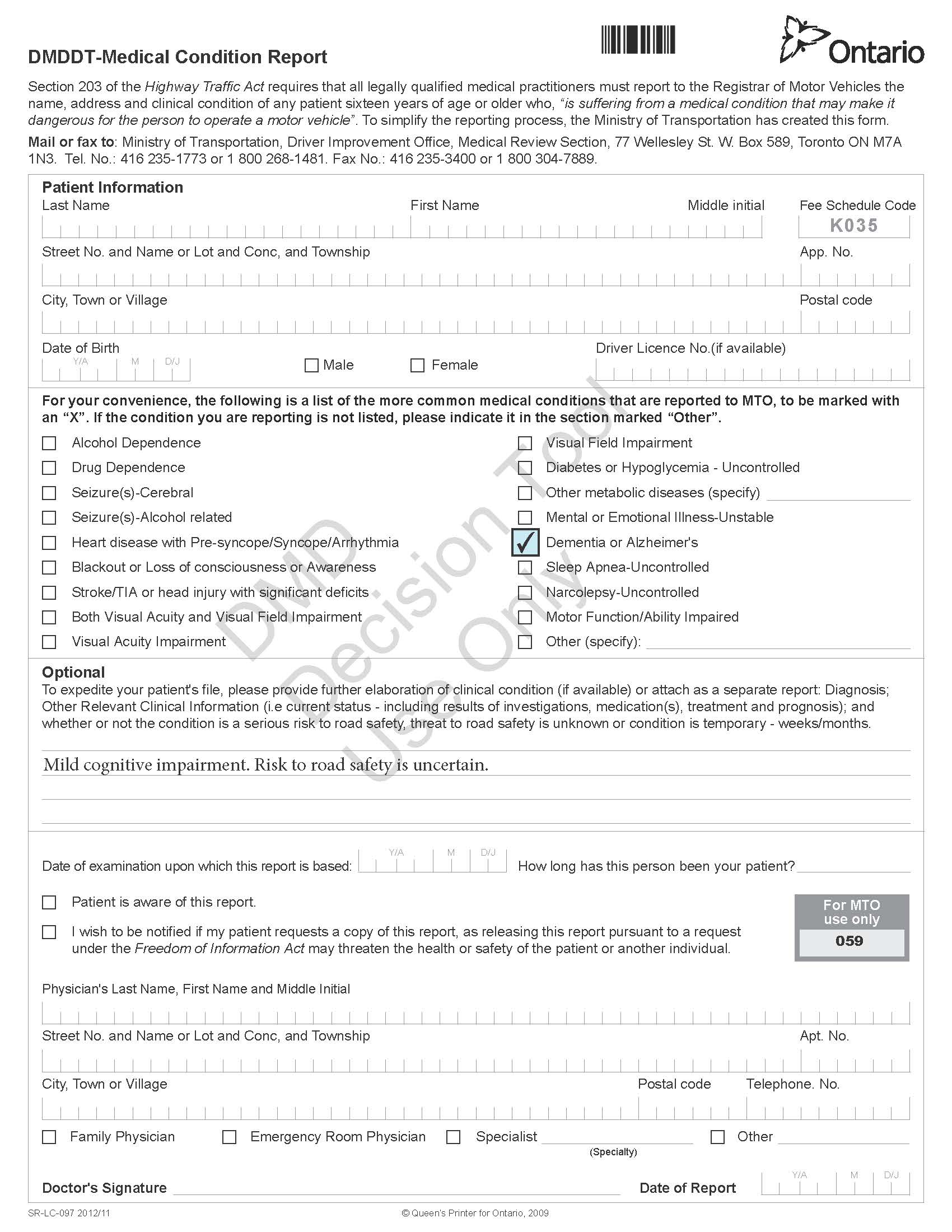

Supplement: Multimedia Appendix 5 [file jmir_v20i5e194_app5.jpg]
