# Supplementary material for: Computer-Based Driving in Dementia Decision Tool With Mail Support: Cluster Randomized Controlled Trial
Source: J Med Internet Res. 2018 May 25;20(5):e194. doi: 10.2196/jmir.9126 (PMC5993977; doi:10.2196/jmir.9126)
Supplement: Multimedia Appendix 6 [file jmir_v20i5e194_app6.pdf]

Multimedia Appendix 6. References contained in newsletters to DD-DT study participants

Barco PP, Baum, CM, Ott BR, Ice S, Johnson A, Wallendorf M, Carr, DB. Driving errors in persons with dementia. J Am Geriatr Soc 2015; 63(7): 1373-1380. PMID: 26140521

Curl AL, Stowe JD, Cooney TM, Proulx CM. Giving up the keys: how driving cessation affects engagement in later life. Gerontologist 2014; 54(3): 423-433. PMID: 23651920

Dommes A, Wu YH, Aquino JP, Pitti-Ferrandi H, Soleille M, Martineau-Fleury S, Samson M, Rigaud AS. Is Mild dementia related to unsafe street-crossing decisions? Alzheimer Dis Assoc Disord 2015; 29(4): 294-300. PMID: 25494367

Ontario Road Safety Annual Report 2011. Toronto: Ontario Ministry of Transportation, Road Safety Policy Office, 2011. http://www.mto.gov.on.ca/english/publications/ontario-road-safety-annual-report.shtml. (Webcitation not available.)

Wernham M, Jarrett PG, Stewart C, MacDonald E, MacNeil D, Hobbs H. Comparison of the SIMARD MD to clinical impression in assessing fitness to drive in patients with cognitive impairment. Can Geriatr J 2014; 17(2): 63-69. PMID: 24883164

Williams AF, Shabanova VI. Responsibility of drivers, by age and gender, for motor-vehicle crash deaths. J Safety Research 2003; 34(5): 527-531. PMID: 14733986

Yi J, Lee HCY, Parsons R, Falkmer T. The effect of the global positioning system on the driving performance of people with mild Alzheimer’s disease. Gerontology 2015; 61(1): 79–88. PMID: 25342271
