# Supplementary material for: Computer-Based Driving in Dementia Decision Tool With Mail Support: Cluster Randomized Controlled Trial
Source: J Med Internet Res. 2018 May 25;20(5):e194. doi: 10.2196/jmir.9126 (PMC5993977; doi:10.2196/jmir.9126)
Supplement: Multimedia Appendix 7 [file jmir_v20i5e194_app7.pdf]

Multimedia Appendix 7. Comparisons between enrolled participants who used the tool and those who did not

Variable	Enrolled Participants who Used the Tool n=38	Enrolled Participants who Did not Use the Tool n=31	Statistic
Intervention group (n,%)<br>Control group (n,%)	21 (60%)<br>17 (50%)	14 (40%)<br>17 (50%)	$\chi^2(1) = 0.697$, $P = .40$
Male physicians (n,%)<br>Female physicians (n,%)	13 (46%)<br>25 (61%)	15 (54%)<br>16 (39%)	$\chi^2(1) = 1.423$, $P = .23$
Rural physicians (n,%)<br>Urban physicians (n,%)	2 (67%)<br>36 (55%)	1 (33%)<br>30 (45%)	Fisher's Exact Test, $P = 1.00$
Hospital-based practice (n,%)<br>Community-based practice (n,%)	23 (61%)<br>15 (48%)	15 (39%)<br>16 (52%)	$\chi^2(1) = 1.017$, $P = .31$
Family physicians (n,%)<br>Specialists (n,%)	17 (50%)<br>21 (60%)	17 (50%)<br>14 (40%)	$\chi^2(1) = 0.697$, $P = .40$

Variable	Enrolled Participants who Used the Tool n=38[a]	Enrolled Participants who Did not Use the Tool n=29	Statistic
Physician years in practice (Mean, SD)[b]	13.750 (9.72)	18.250 (13.18)	F (1,57) = 2.185, $P = .15$
Risk Taking Scale (Mean, SD)[c]	16.84 (4.61)	19.52 (4.79)	F (1,60) = 4.702 $P = .03$
Stress from Medical Uncertainty Scale (Mean, SD) [c]	42.74 (10.74)	37.22 (10.81)	F (1,60) = 3.765 $P = .057$

[a] n=38 participants used the tool, however 2 participants' uses were removed from analysis (see Figure 1)
[b] data missing for n=11 participants
[c] data missing for n=8 participants
