# Supplementary material for: Computer-Based Driving in Dementia Decision Tool With Mail Support: Cluster Randomized Controlled Trial
Source: J Med Internet Res. 2018 May 25;20(5):e194. doi: 10.2196/jmir.9126 (PMC5993977; doi:10.2196/jmir.9126)
Supplement: Multimedia Appendix 8 [file jmir_v20i5e194_app8.pdf]

Multimedia Appendix 8. Tool recommendations compared with participant action

Intervention Group<br>n=114 observations	Participant Reported<br>n (%)	Participant Did Not Report<br>n (%)
Tool Recommendation: Report	32 (28%)	8 (7%)
Tool Recommendation: No Consensus	25 (22%)	34 (30%)
Tool Recommendation: Do Not Report	1 (1%)	14 (12%)

Control Group<br>n=103 observations	Participant Reported<br>n (%)	Participant Did Not Report<br>n (%)
Tool Recommendation: Report	36 (35%)	8(8%)
Tool Recommendation: No Consensus	14 (14%)	23 (22%)
Tool Recommendation: Do Not Report	1 (1%)	21 (20%)
