# Supplementary material for: Computer-Based Driving in Dementia Decision Tool With Mail Support: Cluster Randomized Controlled Trial
Source: J Med Internet Res. 2018 May 25;20(5):e194. doi: 10.2196/jmir.9126 (PMC5993977; doi:10.2196/jmir.9126)
Supplement: Multimedia Appendix 9 [file jmir_v20i5e194_app9.pdf]

Multimedia Appendix 9. Rationale for "Do not Report" by 6 intervention group participants when tool recommended that the patient be reported

Case	Participant	Rationale
1	1	Will have detailed OT driving and cognitive assessment to add to data before informing patient and family. Patient currently not driving and is compliant
2	1	Needs to be cleared by cardiology first - has arrhythmias + ICD
3	2	He is functionally intact and I believe that his low educational status plays a role in his cognitive testing
4	2	She has a mild dementia and I felt a road test would be helpful
5	3	Further investigation is necessary including B12 levels. Also mood may be a factor and lack of sleep. She is on home O2 and there may be times when her sats are low
6	4	Son now has patient's car so she is not driving. Son plans to discuss this with patient and decide if the plan will be to not drive (with my report to [transportation administrator]) vs road test. If patient does not do the road test or chooses not to drive I will report to [transportation administrator]
7	5	She has agreed to stop driving until advised to resume
8	6	Patient was anxious, states she only goes to fitness and doctor's office which are within 1 km
